# Increased expression of *ID2*, *PRELP* and *SMOC2* genes in patients with endometriosis

**DOI:** 10.1590/1414-431X20175782

**Published:** 2017-07-03

**Authors:** F.M. Araujo, J. Meola, J.C. Rosa-e-Silva, C.C.P. Paz, R.A. Ferriani, A.A. Nogueira

**Affiliations:** 1 Departamento de Ginecologia e Obstetrícia, Faculdade de Medicina de Ribeirão Preto, Universidade de São Paulo, Ribeirão Preto, SP, Brasil; 2 Departamento de Genética, Faculdade de Medicina de Ribeirão Preto, Universidade de São Paulo, Ribeirão Preto, SP, Brasil

**Keywords:** Gene expression, Endometriosis, PRELP, SMOC2, ID2, Real-time PCR

## Abstract

Endometriosis is a benign, estrogen-dependent disease with symptoms such as pelvic pain and infertility, and it is characterized by the ectopic distribution of endometrial tissue. The expression of the *ID2*, *PRELP* and *SMOC2* genes was compared between the endometrium of women without endometriosis in the proliferative phase of their menstrual cycle and the eutopic and ectopic endometrium of women with endometriosis in the proliferative phase. Paired tissue samples from 20 women were analyzed: 10 from endometrial and peritoneal endometriotic lesions and 10 from endometrial and ovarian endometriotic lesions. As controls, 16 endometrium samples were collected from women without endometriosis in the proliferative phase of menstrual cycle. Analysis was performed by real-time polymerase chain reaction (PCR). There was no significant difference between gene expression in the endometrium of women with and without endometriosis. The *ID2* gene expression was increased in the most advanced stage of endometriosis and in ovarian endometriomas, the *PRELP* was more expressed in peritoneal lesions, and the *SMOC2* was highly expressed in both peritoneal and endometrioma lesions. Considering that the genes studied participate either directly or indirectly in cellular processes that can lead to cell migration, angiogenesis, and inappropriate invasion, it is possible that the deregulation of these genes caused the development and maintenance of ectopic tissue.

## Introduction

Endometriosis is a benign, estrogen-dependent disease with symptoms such as pelvic pain and infertility; it is characterized by the ectopic distribution of endometrial tissue, particularly in the pelvic peritoneum and ovaries (1). Endometriosis affects 10–15% of women of reproductive age and 35–50% of women with infertility, pelvic pain, or both (2,3).

Endometriosis is believed to occur primarily because of retrograde menstruation and implantation of endometrial cells in the abdominal cavity (4). However, some molecular characteristics seem to favor the onset and progression of ectopic implantation and might explain why only certain women develop the disease (5). In the last decade, several studies on gene expression profiling have demonstrated that many genes are deregulated in endometriosis (6). Previous studies from our group using a method for the screening of differential gene expression have suggested that *ID2*, *SMOC2* and *PRELP* expressions are altered in endometriotic lesions compared to eutopic tissues of patients with endometriosis and of patients without the disease in the early proliferative phase of the menstrual cycle (7).

Endometriomas exhibit certain characteristics that are similar to malignant tumors such as increased growth, vascularization, and tissue invasion. However, tumor characteristics such as monoclonal expansion and genetic abnormalities remain unclear (5). The *ID2* gene (inhibitor of DNA binding 2, a dominant negative helix-loop-helix protein) alters the components of the cell cycle that are normally involved in regulating its progression and overexpression, and seems to make cancer cells resistant to the growth inhibitory effects of various tumor suppressor proteins (8). Given the similarities between endometriosis and cancer, the changes of expression in genes associated with cell adhesion, protein glycosylation, cell invasion, and angiogenesis may affect endometriosis in the same way they affect cancer (9).

The *PRELP* (proline/arginine-rich end leucine-rich repeat protein) gene, which encodes the protein prolargin, belongs to a family of leucine-rich repeat (LRR) proteins (10–12). The involvement of members of this family in collagen fibrillogenesis as well as cellular growth, differentiation, and migration reveal their importance in shaping the extracellular matrix (13).

Alternatively, the *SMOC2* (SPARC-related modular calcium binding 2) gene is mainly expressed in the extracellular matrix and codes for the matricellular protein SMOC2, which is highly expressed during embryogenesis and cicatrization. Matricellular proteins influence a variety of cellular functions, including growth factor signaling, migration, adhesion, cicatrization, angiogenesis, and cell proliferation (14,15).

In the current study, we compared the expression of the *ID2*, *PRELP* and *SMOC2* genes in endometriotic lesions (ovarian and peritoneal) and in the eutopic endometrium of women with and without endometriosis in the proliferative phase of their menstrual cycle. Comparative studies of gene expression between these tissues are important in order to identify whether deregulated gene expression is already present in the endometrium of these women or if this expression is only altered by the peritoneal environment (when this tissue falls into the cavity) and, thus, acquires the potential to develop into endometriotic lesions.

## Material and Methods

The participants were recruited at the tertiary hospital of the Faculdade de Medicina de Ribeirão Preto (FMRP), Universidade de São Paulo, Ribeirão Preto, SP, Brazil. The study was approved by the Research Ethics Committees of FMRP, and all participants gave written informed consent.

### Samples

A case-control study was conducted on women with and without endometriosis in the proliferative phase of the menstrual cycle. Twenty patients with a laparoscopic and histological diagnosis of endometriosis were selected. The subjects were 18 to 40 years old, not menopausal, had not had any hormone therapy for at least 6 months before sample collection, and had no other reproductive disorders or any tumors. The stage of endometriosis was determined according to the classification of the American Society for Reproductive Medicine (16). Paired tissue samples from 20 women were analyzed: 10 were from eutopic endometrium and peritoneal endometriotic lesions and 10 were from eutopic endometrium and ovarian endometriomas. Only one lesion per patient was collected. All ovarian lesions were composed exclusively of cystic endometriotic lesions, since the capsule of the endometrioma was surgically removed from the ovary. For peritoneal lesions, surgical dissection was performed without surgical margin so that the tissue was basically composed of endometriotic lesion. Half of the lesions were used for histological analyses and the other half were processed for RNA extraction. Of the 20 biopsies from ectopic endometria, 10 were peritoneal lesions (6 red and 4 black), three in stage I, four in stage II, two in stage III, and one in stage IV; the other 10 were ovarian lesions (ovarian endometriomas), including four in stage III and six in stage IV. Eutopic endometrium was collected with a Novak curette (AESCULAP, USA) during laparoscopy.

The control group consisted of 16 women of reproductive age (18–40 years of age) without endometriosis, fibrosis, pelvic adhesions, or infertility.

These women were subjected to laparoscopy for tubal ligation with laparoscopic confirmation of the absence of endometriotic lesions. Data collected were standardized according to each woman's menstrual cycle in the proliferative phase, which was confirmed by histological criteria. The endometrial biopsy was collected with a Novak curette during the surgical procedure.

The samples were stored in a freezer at –80°C in a cryotube containing RNAlater solution (Ambion Life Technologies, UK) for RNA preservation.

### RNA extraction and cDNA synthesis

The samples were washed in 1×PBS (8.50 g/L NaCl, 1.11 g/L Na_2_HPO_4_, 2.81 g/L Na_2_HPO_4_.12H_2_O, 0.20 g/L KH_2_PO_4_, pH 7.0) to remove the RNAlater solution. Next, the total RNA (50 mg tissue) was extracted with TRIzol® reagent (Invitrogen Life Technologies, UK) and treated with DNase I (Invitrogen Life Technologies) according to the manufacturer's instructions. The RNA integrity and purity was confirmed by the presence of the 28S and 18S ribosome bands and absence of DNA genomic band when analyzed by 1% agarose gel electrophoresis with 1×MOPS buffer. The total RNA concentration was determined by spectrophotometry (NanoDrop 2000c, Thermo Scientific, USA) at 260 nm. The extracted total RNA was stored in a freezer at –80°C for subsequent use. One microgram of total RNA from each sample was reverse-transcribed using the HighCapacity cDNA Transcription Kit (Applied Biosystems Life Technologies, UK), according to the manufacture’s instructions.

### Real-time polymerase chain reaction (PCR)

The probes and primers for the *ID2* (Hs00747379_m1), *PRELP* (Hs00160431_m1) and *SMOC2* (Hs00405777_m1) genes, and the reference genes for the reaction, *GAPDH* (glyceraldehyde-3phosphate dehydrogenase) (Hs99999905_ml) and *ACTB* (actin, beta) (Hs99999903_m1), were obtained using Assay-on-Demand™ Gene Expression Products (Applied Biosystems, UK). Quantification was performed on an ABI PRISM™7500FAST Sequence Detection System (Applied Biosystems). Real-time PCR was performed in duplicate for each sample according to the following conditions: 10 µL of TaqMan¯ Universal PCR Master Mix (2×; Applied Biosystems), 1 µL of TaqMan¯ Gene Expression Assay Mix (20×; Applied Biosystems), and 9 µL of diluted cDNA in a final reaction volume of 20 μL. The reaction conditions consisted of 50°C for 2 min, 95°C for 10 min, 40 cycles at 95°C for 15 s, and 60°C for 1 min.

In order to confirm the purity of the RNA, a pool of the total RNA (without cDNA) from all samples was done, and then real-time PCR was performed for this "sample" using the *GAPDH* probe (Hs99999905_ml). This probe can amplify both DNA and cDNA of the target.

The relative quantification (RQ) of genes was calculated for each sample according to the 2-delta delta Ct (2^-ΔΔ*C*T^) method. A pool of cDNA containing equal quantities of endometrium samples obtained from women without endometriosis (control group) was used to calibrate the reactions. *GAPDH* and *ACTB* genes were used as reference genes to normalize the reactions.

### Statistical analysis

The gene expression variables (RQ) were log_10_ transformed [log10(RQ+10)]. Logarithmic transformation was necessary because one of the assumptions (linearity) in the linear model analysis was not satisfied. The transformed data are shown in the Figures (means±SD). Statistical analyses were performed using SAS software (2002-2003, SAS Institute Inc., USA). We applied the Welch's unpaired ANOVA to compare: A, the endometrium of women with and without endometriosis, and B, stages I/II with stages III/IV. Paired *t*-tests were used to compare the gene expression means obtained from: C, the eutopic and ectopic endometrium of 10 women with peritoneal endometriosis; D, the eutopic and ectopic endometrium of 10 women with ovarian endometriomas, and E, the eutopic and ectopic endometrium of women with endometriosis (peritoneal lesions in addition to ovarian endometriomas). All tests were performed using GLM procedures. The study samples were sufficient for the analyses with a power (1-β) of at least 0.80 and a level of significance of (α)=0.05.

## Results

There was no significant difference between the expression of the *ID2*, *PRELP* and *SMOC2* genes in the endometrium of women without endometriosis and in the eutopic endometrium of women with endometriosis.

**Figure 1.**
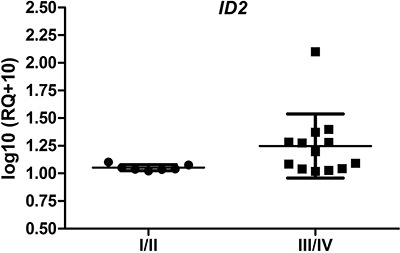
Expression levels of *ID2* gene in endometriosis I/II *vs* III/IV. Horizontal lines indicate medians and interquartile range.

Comparison of stages I/II (initial endometriosis) with stages III/IV (advanced endometriosis) in unpaired samples revealed a significant difference for only the *ID2* gene (1.05±0.03 *vs* 1.25±0.29, P=0.03), which showed higher expression in advanced endometriosis (Figure 1).

**Figure 2.**
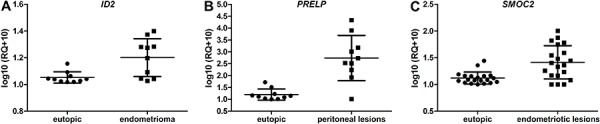
Expression levels of *ID2* (*A*), *PRELP* (*B*) and *SMOC2* (*C*) genes in the eutopic endometrium and endometriotic lesions (endometrioma, peritoneal lesions and endometriotic lesions) of women with endometriosis. Horizontal lines indicate medians and interquartile range.

Regarding the comparison of peritoneal and ovarian lesions, when studied separately with the paired eutopic endometrium, the *ID2* gene was expressed more in endometrioma lesions (1.05±0.04 *vs* 1.20±0.14, P=0.002; Figure 2A), while the *PRELP* gene was expressed more in peritoneal lesions (1.2±0.23 *vs* 2.74±0.95, P=0.003; Figure 2B). There was no difference in any other comparison.

In the comparative analysis of the lesions as a whole (peritoneal and ovarian), only the *SMOC2* gene revealed significant differences (1.12±0.11 *vs* 1.41±0.31, P<0.001) and showed higher expression in ectopic tissues (Figure 2C).

## Discussion

In 2010, our group analyzed genes differentially expressed on a large-scale basis in the eutopic and ectopic endometrium of patients with endometriosis (7). Our data suggested that *ID2*, *SMOC2* and *PRELP* expressions are altered in endometriotic lesions. In the current study, we used real-time PCR to validate those data. A significant difference was found in the expression of the *ID2*, *PRELP* and *SMOC2* genes, which had higher expression levels in endometriotic lesions.

ID proteins alter the components of the cell cycle that are normally involved in regulating progression (8,17). Another gene also involved in cell cycle regulation is the retinoblastoma (*RB*) gene, which was the first tumor suppressor to be cloned and it negatively regulates the cell cycle (18). Overexpression of *ID2* renders pRb inactive and also nullifies the inhibitory activity of the cell cycle proteins p107 and p130, which are related to pRb (19,20).

Goumenou et al. (21) analyzed possible differences in protein expression of p16, pRb, and cyclin D1 in endometriomas and adenomyomas using immunohistochemistry techniques. The p16 protein was present in 77% of adenomyomas and 15% of endometriomas, while pRb was detected in 28% of the endometriomas analyzed but not in adenomyomas. Cyclin D1 was not found in the analyzed tissues. This study demonstrated that p16 and pRb play important roles in regulating cell growth in adenomyomas and endometriomas, respectively. Our results showed higher expression of the *ID2* gene in ovarian endometriomas and, considering that its overexpression can inhibit pRb's capacity as a tumor suppressor in cancer cells, it is possible that it also behaves in this way in endometriosis.

The *PRELP* and *SMOC2* genes are involved in the modeling of the extracellular matrix by stimulating the proliferation and migration of endothelial cells as well as angiogenesis activities (13,15). It was previously suggested that, in endometriosis, the proteins from the extracellular matrix might be responsible for disturbance in physiological functions such as endometrial secretion, implantation, and menstruation (22). Other studies found high expression of these proteins in the ectopic endometrium, which could lead to the development of endometriosis (23).

In the analysis including all the endometriosis lesion, the *PRELP* gene presented increased expression in peritoneal lesions compared with the eutopic endometrium of women with peritoneal endometriosis, and the *SMOC2* gene was expressed more in the lesions (endometriotic and peritoneal) compared with the eutopic endometrium of women with endometriosis.

Previous studies identified 22 microRNAs that were differentially expressed in ectopic and eutopic endometrium of paired samples from women with and without endometriosis (24). Zhao et al. (25) examined the presence of SNPs near these miRNA targets. Among the 102 SNPs analyzed in different genes, the SNP rs7542469 of the *PRELP* gene was found in the control group but not in women with endometriosis. Our results were in disagreement, as we found increased expression of this gene in peritoneal lesions. This phenomenon could be related to changes in the peritoneal environment, which are responsible for the transformation of the endometrium into an endometriotic lesion.

Our results regarding the *SMOC2* gene corroborate the findings by Eyster et al. (9), who analyzed the pattern of gene expression in the ectopic and eutopic endometrium of 11 patients in order to identify possible gene families involved in endometriosis. In that screening, which used microarrays of the 717 analyzed genes, the *SMOC2* gene, among others, had a higher expression rate in the ectopic than in the eutopic endometrium. Since real-time PCR is the gold standard for the quantification of gene expression, it is possible that *SMOC2* may be related to or participate directly in the development of endometriosis.

Our results indicate that the expression of the *ID2*, *PRELP* and *SMOC2* genes were influenced by the peritoneal environment and intrinsic mechanisms of endometriotic lesions themselves, which are shaped by their maintenance and evolution. This may change depending upon the site of implantation (ovary or peritoneal) and severity of the lesion. Additionally, the expression of *ID2*, *PRELP* and *SMOC2* genes is similar between the normal endometrium and the eutopic endometrium of women with endometriosis.
